# ZooCNN: A Zero-Order Optimized Convolutional Neural Network for Pneumonia Classification Using Chest Radiographs

**DOI:** 10.3390/jimaging11010022

**Published:** 2025-01-13

**Authors:** Saravana Kumar Ganesan, Parthasarathy Velusamy, Santhosh Rajendran, Ranjithkumar Sakthivel, Manikandan Bose, Baskaran Stephen Inbaraj

**Affiliations:** 1 Department of Electronics and Communication Engineering, Karpagam College of Engineering, Coimbatore 641032, India; 2 Department of Computer Science Engineering, Karpagam Academy of Higher Education (Deemed to Be University), Coimbatore 641021, India; sarathy.vp@gmail.com (P.V.); santhoshrd@gmail.com (S.R.); ranjithbca27@gmail.com (R.S.); selvamanikandan784@gmail.com (M.B.); 3 Department of Food Science, Fu Jen Catholic University, New Taipei City 242062, Taiwan

**Keywords:** convolutional neural network, zero-order optimization, hyperparameter optimization, pneumonia classification, chest X-ray images

## Abstract

Pneumonia, a leading cause of mortality in children under five, is usually diagnosed through chest X-ray (CXR) images due to its efficiency and cost-effectiveness. However, the shortage of radiologists in the Least Developed Countries (LDCs) emphasizes the need for automated pneumonia diagnostic systems. This article presents a Deep Learning model, Zero-Order Optimized Convolutional Neural Network (ZooCNN), a Zero-Order Optimization (Zoo)-based CNN model for classifying CXR images into three classes, Normal Lungs (NL), Bacterial Pneumonia (BP), and Viral Pneumonia (VP); this model utilizes the Adaptive Synthetic Sampling (ADASYN) approach to ensure class balance in the Kaggle CXR Images (Pneumonia) dataset. Conventional CNN models, though promising, face challenges such as overfitting and have high computational costs. The use of ZooPlatform (ZooPT), a hyperparameter finetuning strategy, on a baseline CNN model finetunes the hyperparameters and provides a modified architecture, ZooCNN, with a 72% reduction in weights. The model was trained, tested, and validated on the Kaggle CXR Images (Pneumonia) dataset. The ZooCNN achieved an accuracy of 97.27%, a sensitivity of 97.00%, a specificity of 98.60%, and an F1 score of 97.03%. The results were compared with contemporary models to highlight the efficacy of the ZooCNN in pneumonia classification (PC), offering a potential tool to aid physicians in clinical settings.

## 1. Introduction

Pneumonia is a severe respiratory infection that impairs lung function, often caused by bacteria or viruses filling the alveoli with pus and fluid. This accumulation makes breathing difficult and reduces oxygen intake. The severity of pneumonia can range from mild to life-threatening, and it is responsible for 14% of all deaths in children under 5 years old. In 2019, pneumonia was responsible for 740,180 deaths in children under five, making it the leading infectious cause of death in this age group. Other at-risk populations include older adults and individuals with pre-existing health conditions [1].

Pneumonia is commonly classified into three types: Community-Acquired Pneumonia (CAP), Hospital-Acquired Pneumonia (HAP), and Ventilator-Associated Pneumonia (VAP), with CAP being the most prevalent [1]. Various diagnostic tools are employed to detect pneumonia, including CXR, Computed Tomography (CT), and Magnetic Resonance Imaging (MRI). Among these, CXRs are widely considered the most effective and efficient tool for pneumonia detection. Compared to CT scans, CXRs are less expensive, involve reduced radiation exposure, and provide faster results. While MRI offers superior soft tissue contrast, it is costlier and less accessible than CXR [2,3]. Consequently, CXRs are a simple and economical diagnostic method that is routinely used for PC. Physicians evaluate CXR images to identify the structural changes or deviations caused by pneumonia. However, accurately interpreting CXR images requires expertise, which is challenging when treating patients in LDCs due to the shortage of physicians relative to the global pneumonia burden [4]. This shortage highlights the need for automated systems to aid physicians in diagnosing pneumonia more efficiently.

Convolutional Neural Networks (CNNs), a type of Deep Learning algorithm, have been proposed as a promising tool for this task. CNNs can automatically extract features from medical images, making them suitable for classifying diseases based on radiographic data. Recent advancements in CNNs have greatly improved pneumonia classification. For instance, X-ODFCANet introduced an omni-dimensional dynamic convolution feature coordinate attention network, improving classification accuracy by 3.77% compared to ResNet18 through feature coordination attention modules [5]. Similarly, the Efficient PM Multisampling approach tackles noise and class imbalances using Perona–Malik multisampling and Generative Adversarial Networks (GANs), achieving a 96% accuracy rate without overfitting [6].

Stacked Ensemble Learning, which combines deep learning features and a stacking classifier, achieved 98.3% accuracy and 99.29% precision in pediatric pneumonia diagnosis [7]. Additionally, an ensemble of EfficientNetv2-L and YOLO for region-of-interest localization provided a mean average precision score of 0.617 on public datasets [8]. CNN-based diagnostic tools have also demonstrated significant improvements. A two-step CNN pipeline has shown high sensitivity (91.8% to 95.8%) and specificity (96.6% to 97.8%) in differentiating pneumonia, Acute Respiratory Distress Syndrome (ARDS), and normal lungs [9].

ResNet and DenseNet architectures have performed well in medical image classification tasks, including pneumonia and COVID-19 detection, with DenseNet-201 also showing promise in malaria classification [10,11]. While architectures like VGG16 and VGG19 have been widely used, they tend to underperform compared to more advanced models like DenseNet and ResNet. However, MobileNet, despite being simpler, has shown satisfactory results in certain lightweight applications [12]. Metrics such as accuracy, sensitivity, and specificity are essential, and CNN models have achieved over 94% accuracy in tasks like ventricular fibrillation detection [13]. However, cross-dataset robustness remains a challenge, which indicates the need for additional adjustments to maintain performance across varied datasets [14].

Furthermore, advanced methods such as simulated annealing particle swarm optimization have been integrated into CNN models for pneumonia classification, optimizing hyperparameters without relying on gradient information, which is crucial for large datasets like the Kaggle Pneumonia Chest X-ray Images dataset [15,16]. Moreover, Zeroing Neural Dynamics (ZND) has been proposed to accelerate optimization in CNNs by transforming gradient information [17]. However, these methods must balance computational complexity with real-world performance [18]. The aim of this work is to improve the performance of an Automated Detection System (ADS) in healthcare, particularly in PC. The contemporary CNN architectures present a trade-off between the use of resource-intensive accuracy models and the compromises regarding classification made when using efficient models. This article aspires to address a critical question in the development of DL models for CXR analysis: “is a balance between classification accuracy and computational efficiency feasible through the optimization of CNN architecture for the classification of X-ray images into three classes—NL, BP and VP”? This study aims to achieve the following goals:

I. To optimize a CNN model for PC with high accuracy while minimizing computational costs.
II. To dynamically optimize the hyperparameters during the training of CNNs for PC.
III. To develop strategies for reducing overfitting in CNN models, especially when trained on imbalanced datasets.

## 2. Materials and Methods

**Figure 1:**
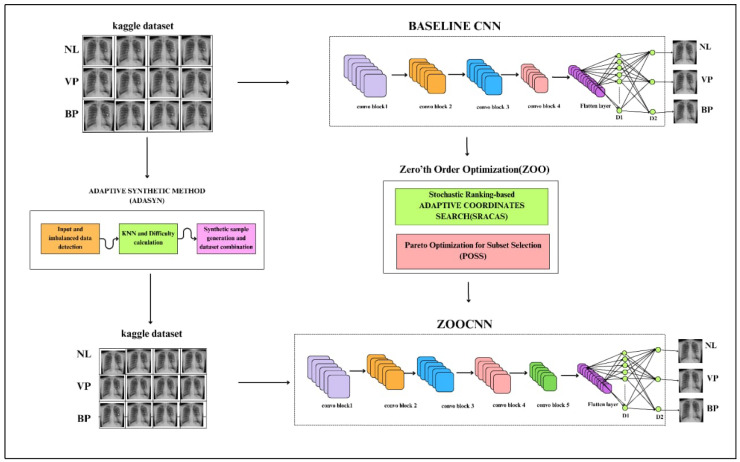
Framework for imbalanced CXR image classification using ZOO and ADASYN.

This methodology begins with the preprocessing of an imbalanced Kaggle CXR dataset using the Adaptive Synthetic (ADASYN) method to generate synthetic samples to obtain a balanced dataset, as shown in Figure 1. A baseline CNN (CNN:I) with four convolutional blocks is first trained on the original dataset. To optimize the CNN:I’s performance, a ZOO strategy is employed, leveraging Stochastic Ranking-based Adaptive Coordinate Search (SRACOS) and Pareto Optimization for Subset Selection (POSS) to obtain the ZooCNN. The optimized architecture, ZooCNN, incorporates a fifth convolutional block, followed by dense layers, to provide PC for NL, VP, and BP.

### 2.1. Operational Workflow of CNN

The CNN architectures in this article were fed with CXR images with a size of 224 × 224, with one channel (monochrome). The input shape was defined as (ni, 224 × 224), where ni is the batch size. The convolutional layers transformed the spatial dimensions, yielding ‘feature maps’, and the output shape of the convolutional layer was calculated using the number of filters (nf), the kernel size (K), stride (S), and padding (P).

(1) $$output\;shape\;of\;convolutional\;layer=\left(\frac{Input\;Size-Kernel\;Size}{Stride}\right)\times \mathrm{number}\;\mathrm{of}\;\mathrm{filters}$$

The convolutional operation is defined by

(2) $$Y_{i}=ReLU\left(W_{i}\times X_{i-1}+b_{i}\right)$$

where $Y_{i}$ represents the filters of size m × m applied to input X_i−1_ (the output of the previous layer) and bi is the bias term. The operation × denotes the convolution and Y_i_ is the output feature map.

Max pooling layers were applied to reduce the spatial dimensions, defined mathematically as follows:

(3) $$Y_{pool}=\underset{pool\;window}{max}\left(Y_{i}\right)$$

This operation downsamples the input by taking the maximum value within each pooling window with a size of 2 × 2. The output size after pooling is calculated as follows:

(4) $$Pooled\;Output\;Size=\left(\frac{Convolved\;Output\;Size}{2}\right)$$

The flattening layer transforms the 2D feature maps into a 1D vector Z, such that

(5) $$Z=Flatten(Y_{last\;conv})$$

where $Y_{last\;conv}$ is the output of the last convolutional layer.

The fully connected layer then performs the following equation:

(6) $$Z_{dense}=ReLU(W_{dense}Z+b_{dense})$$

where $W_{dense}$ is the weight matrix of the dense layer and $b_{dense}$ is the bias vector.

The final output layer applies the Softmax function to produce a probability distribution for each class: NL, BP, and VP.

(7) $$P\left(y=j|x\right)=\frac{\exp\left(Z_{dense}^{T}W_{j}+b_{j}\right)}{\sum_{k-1}^{3}\exp\left(Z_{dense}^{T}W_{k}+b_{k}\right)}$$

where P(y = j|x) is the probability of class j given input x, W_j_, b_j_, and the parameters corresponding to class j.

### 2.2. CNN: I Architecture

This article uses a baseline DL model, CNN: I, designed exclusively for PC on the Kaggle CXR images dataset, and its architectural details are presented in Table 1. The number of kernels in the first, second, third, and fourth convolutional layers are 16, 32, 64 and 128, respectively. The values of input shape and output shape and the hyperparameter values are presented in Table 2 for CNN:I.

Owing to the large number of hyperparameters, CNN:I’s implementation increases the risk of overfitting, improves the model’s generalization ability, requires complex hyperparameter finetuning, and leads to a longer training time. To address these drawbacks, ZOO was applied to CNN:I to develop ZooCNN.

### 2.3. ZooPT Framework

The ZooPT’s framework allows for derivative-free optimization, addressing the issues associated with the traditional gradient methods in hyperparameter tuning. The hyperparameter space of a CNN is a combination of continuous parameters, namely the learning rate, dropout rate, and discrete parameters that include the number of layers and the number of filters.

#### Mathematical Formulation

1. Search Space Definition

**Figure 2:**
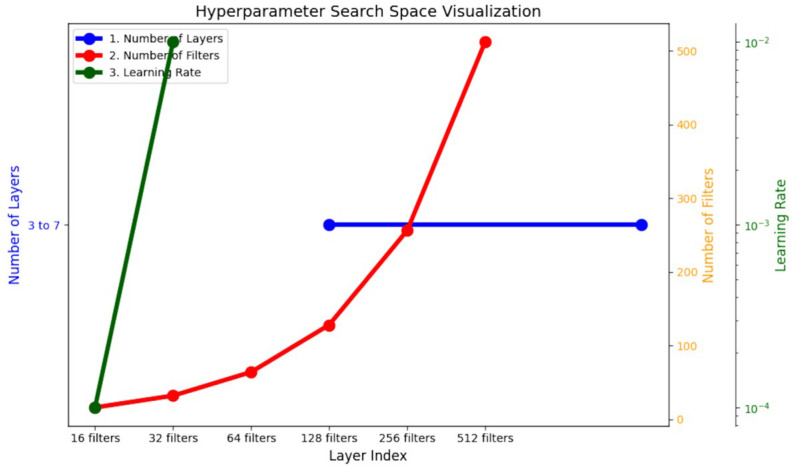
Hyperparameter search space for the CNN.

Mathematically, the problem of hyperparameter optimization for CNN can be formulated as an optimization problem in a high-dimensional search space, S, as shown in Figure 2.

(8) $$\Theta =\{{l,f}_{1},f_{2},\ldots ,f_{n},\eta ,{l,d}_{1},d_{2},\ldots ,d_{n}\}$$

The search space for the CNN optimization is defined as follows:

i. Number of Filters ${(f}_{i}$): Each convolutional layer has $f_{i}$, a discrete set of the number of possible filter sizes: $f_{1}\in \left\{16,64\right\}$, $f_{2}\in \left\{32,128\right\}$, $f_{3}\in \left\{64,256\right\}$, $f_{4}\in \left\{16,512\right\}$, $f_{5}\in \left\{32,256\right\}$.
ii. Number of Layers (l) varies from three to seven, incorporating the total number of convolutional layers in a network: $\;d\in \left\{3,7\right\}$.
iii. Learning Rate (η): A hyperparameter that is continuous by nature and regulates the step size in the gradient descent process: $\eta \in \left\{0.1,10^{-3}\right\}$.
iv. Dropout rate specifies the fraction of neurons to drop: $d\in \left\{3,7\right\}$.

2. Optimization in Various Spaces with SRACOS

The SRACOS optimization aims to minimize the validation loss $L\left(\Theta \right)$, a function of the hyperparameter space Θ. Mathematically, the validation loss is defined as follows:

(9) $$\Theta^{*}=arg\underset{\left\{\Theta \right\}]}{\;min}L\left(\Theta \right)$$

where $L\left(\Theta \right)$ denotes the expected validation loss, averaged over multiple evaluations to account for noise.

The optimization algorithm operates through an iterative refinement of the search space. For every iteration t, a set of candidate configurations is sampled. Then, each candidate is trained using a CNN:I on the Kaggle CXR image dataset and its validation loss is calculated. The best-performing configuration in each iteration is modeled as follows:

(10) $$\Theta_{t+1}\sim p\left(\Theta |L\left(\Theta_{t}^{*}\right)<L\left(\Theta_{t}^{i}\right)\right)$$

(11) $${\Theta^{*}}_{t}=arg\underset{\left\{i\right\}}{\;min}L({\Theta^{*}}_{t}^{i})$$

This influences the sampling distribution of the next iteration, directing the process to identify the configuration with the lowest validation loss.

3. *Optimization with POSS*

The dropout selection made by POSS is based on the impact of the inclusion or exclusion of any layer on the CNN model’s performance. This is mathematically defined as $d_{j}\in \left\{0,1\right\}$, $d_{j}=1$ for exclusion and $d_{j}=0$ for inclusion of the j—th layer.

(12) $$\Theta^{*}=arg\underset{\left\{i\right\}}{\;min}E(L\left(\Theta^{*}\right|d_{j})$$

4. *Dimensionality reduction:*

The CNN hyperparameter search space can be very high-dimensional, especially considering the large quantity of layers, filter sizes, and learning rates that need to be optimized. To mitigate this, ZOO combines random embedding methods with the following transformation of the high-dimensional search space into a lower-dimensional sub-space:

(13) $$\Phi :S\to S^{\prime }$$

S is the original search space and $S^{\prime }$ is the reduced subspace. An optimization is then performed to enable a more efficient exploration of the search space:

(14) $$\Theta^{*}=arg\underset{\Theta \in s^{\prime }}{\;min}L\left(\Theta \right)$$

Therefore, random embedding projects the high-dimensional search space, S, to subspaces of lower dimensionality, $S^{\prime }.$ The optimization inside $S^{\prime }$ will make the search become on the Kaggle Pneumonia dataset more effective.

1. The article optimizes a modified objective function that incorporates depth penalty and complexity terms into the ZooCNN model.

2. Various convolutional layer configurations are explored, including different kernel sizes and incremental increases in the number of kernels, to enhance feature extraction and capture complex patterns in the data.

3. In the dense layers, the number of units is reduced to prevent overfitting and dropout rates are finetuned to improve the generalization performance.

4. A population-based search strategy is employed with a specified number of candidate models, adjusting the exploration-to-exploitation ratio over time to focus progressively on the most promising configurations.

5. The optimization process involves multiple iterations with a defined step size for optimal convergence, and early stopping is applied to prevent overfitting during training.

Table 3 presents the ZooPT optimization attributes for iterating compared to CNN:I, refining the search space to develop a ZooCNN with optimized hyperparameters.

The iterative ZOO on CNN:I resulted in a ZooCNN with optimized network parameters and hyperparameters. The architectural details of the ZooCNN are presented in Table 4 and the values of input shape, output shape, and hyperparameters are presented in Table 5. The number of kernels in the first, second, third, fourth, and fifth convolutional layers are 32, 64, 128, 256, and 512, respectively.

## 3. Results and Discussion

### 3.1. Dataset Description

**Figure 3:**
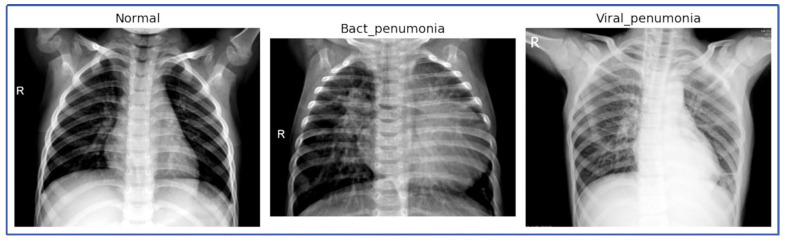
CXR images of NL, BP, and VP.

Figure 3 presents a series of CXR images categorized into three groups: NL, BP, and VP. The images reveal clear distinctions between the different conditions:

1. Normal: The images labeled as ‘normal’ depict clear lung fields without any significant opacities or consolidations. The bronchial and vascular structures are visible and consistent with normal chest radiographs, which serve as a baseline comparison against the pneumonia-affected lungs.
2. Bacterial Pneumonia: Several images labeled as ‘Bact_pneumonia’ exhibit prominent consolidation, with areas of opacity that suggest alveolar filling, which is characteristic of bacterial pneumonia. These radiographic findings are consistent across multiple images, highlighting the typical presentation of bacterial pneumonia.
3. Viral Pneumonia: The ‘viral_pneumo’ images demonstrate more diffuse patterns, with less pronounced opacities compared to bacterial pneumonia. The images show peribronchial thickening and interstitial markings, which align with the expected radiological signs of viral infections.

**Figure 4:**
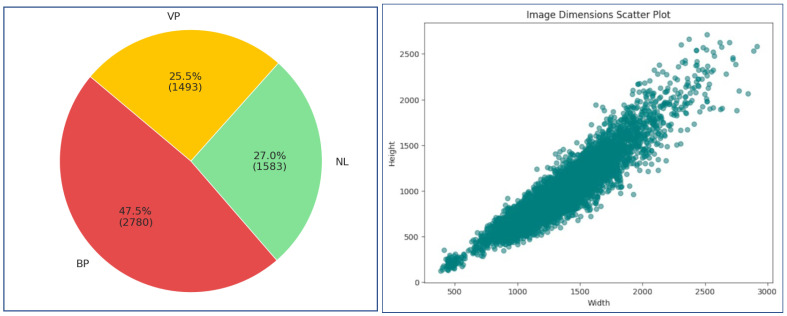
Analysis of Kaggle CXR images dataset: distribution and correlation patterns.

**Figure 5:**
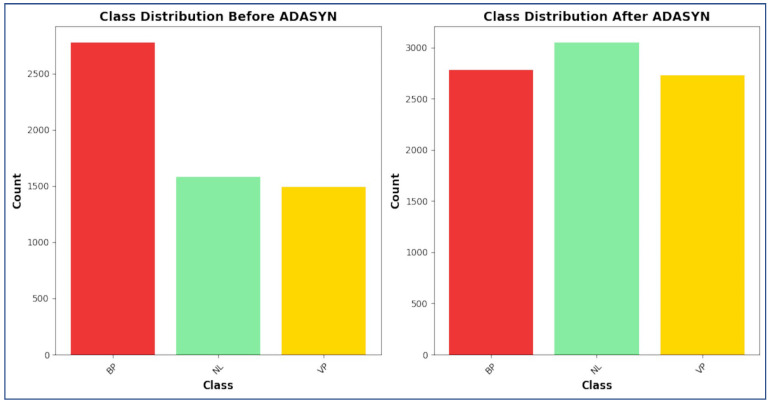
Class distribution before ADASYN (**left panel**) and class distribution after ADASYN (**right panel**).

Figure 4 presents a pie chart displaying the class distribution in the Kaggle CXR dataset, depicting three key categories: BP (47.5%, 2780 images), Normal (27.0%, 1583 images), and VP (25.5%, 1493 images). This distribution highlights a significant class imbalance, with BP comprising almost half of the dataset, while Normal and VP cases represent roughly a quarter each. This imbalance in class representation is a common issue in medical imaging datasets, particularly for pneumonia diagnoses, as indicated by several studies. The scatter plot in Figure 5 illustrates the relationship between image width and height, revealing a strong positive correlation between the two variables. A statistical description of the images is presented in Table 6. These statistics highlight the variability in image dimensions, which impacts subsequent feature extraction and classification.

### 3.2. Dataset Balance Restoration Through the Application of ADASYN

The dataset used in the ChxCapsNet [19] had a similar distribution bias towards pneumonia-related cases, with normal images being underrepresented. Similarly, the CX-DaGAN model, designed for domain adaptation in pneumonia diagnosis [20], utilized a dataset in which pneumonia (both bacterial and viral) were present in a higher proportion compared to normal cases. Class imbalances tend to skew the performance of DL models, potentially leading to a bias toward the majority class. The augmentation of minority classes or implementation of class-weighted losses significantly improves multi-class classification accuracy in lung disease detection tasks [21]. In other publicly available datasets, similar trends of class imbalance are observed, though bacterial pneumonia tends to dominate in most chest X-ray datasets used for pneumonia detection tasks. Therefore, addressing this imbalance through data augmentation or the oversampling of minority classes is critical for model generalization.

Figure 5 displays the class distribution before and after applying the ADASYN technique to the Kaggle CXR image dataset. In the chart on the left, the dataset exhibits a clear imbalance, where Class 0 (BP) is the majority class, with over 2500 samples, while Class 1 (Normal) and Class 2 (VP) have fewer samples, indicating a substantial minority class imbalance. After applying ADASYN (right panel), the distribution is much more balanced. ADASYN generated synthetic samples for the minority classes (Class 1 and Class 2), bringing their sample counts closer to those of the majority class, with the counts for all three classes approaching 3000. This balancing of class distributions ensures that the ZooCNN model will have a more equitable exposure to all classes, reducing the potential bias toward the majority class.

**Figure 6:**
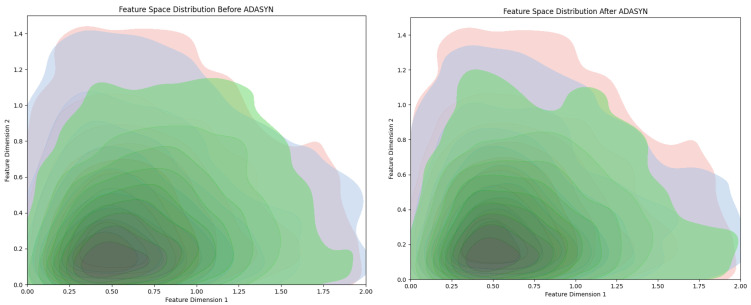
Feature space distribution before ADASYN (**left panel**) and after ADASYN (**right panel**).

Both ADASYN and SMOTE aim to balance class distribution by generating synthetic data for minority classes. The use of SMOTE in their lung disease classification task boosted accuracy and reduced bias towards the majority class [22]. However, ADASYN differs by focusing on generating more samples in areas in which the model has more difficulty distinguishing between classes, thus adapting to the data complexity more effectively [23]. In comparison, SMOTE generates samples uniformly across the minority class, without adapting to the local distribution of samples. While SMOTE can still effectively balance classes, ADASYN may offer more nuanced improvements in highly imbalanced datasets, as shown in Figure 6. Random oversampling duplicates existing minority class samples, which can lead to overfitting since the model sees the same examples multiple times. In contrast, ADASYN generates new synthetic samples, introducing variability and reducing the likelihood of overfitting [24].

### 3.3. CNN: I Hyperparameter Finetuning Using ZooPT

ZooPT was employed to optimize a CNN:I, including the fine-tuning of filter sizes, model complexity, dropout, and learning rate, which resulted in the development of the ZooCNN.

#### 3.3.1. Filter Sizes and Depth

ZooPT increased the filter sizes of several convolutional layers, such as expanding the first Conv2D layer’s filters from 16 to 32 and the fourth Conv2D layer’s from 128 to 256. Larger filter sizes allow the CNN to capture more complex spatial patterns in CXR images, which is critical for detecting pneumonia’s subtle manifestations. By enhancing the network’s capacity for both low-level and high-level feature extraction, the depth of the CNN was increased by approximately 310% when distinguishing between normal and pneumonia-affected lung tissue, which is crucial for identifying minor texture and density changes in X-rays.

#### 3.3.2. Model Complexity and Computational Load

The ZooPT optimization reduced the model’s complexity through architectural modifications and hyperparameter reduction, as shown in Table 7, thereby significantly lowering the computational costs and training complexity.

#### 3.3.3. Parameter Reduction

The ZooCNN achieved a significant reduction in parameters through the dynamic optimization of hyperparameters, such as the number of filters and layers. This optimization decreases the total parameter count from approximately 12.94 million in the baseline CNN to 3.17 million in the ZooCNN, as shown in Table 6. This 72% reduction in parameters lowers the memory requirements but also reduces the training complexity.

#### 3.3.4. Architecture Optimization

In the baseline CNN (denoted as CNN:I), the inclusion of two dense layers with 512 units each results in a high parameter count of approximately 12.94 million, increasing model complexity and elevating the risk of overfitting. In contrast, the ZooCNN reduces the dense layers to 128 units, focusing on obtaining an efficient feature combination while substantially lowering the parameter count to 3.17 million. This streamlined architecture reduces model complexity, enabling faster convergence during training and requiring fewer epochs.

The reduced complexity of the ZooCNN minimizes the GPU and CPU processing demands per epoch, leading to shorter training times and reduced overall computational resource consumption. These improvements are detailed in Table 8, which compares training complexity and efficiency between the baseline CNN and the ZooCNN.

#### 3.3.5. ZooCNN’s Computational Efficiency

The following metrics were used to measure the computational efficiency of the ZooCNN and substantiate its ability to reduce computational costs: training duration and memory usage. Training duration was recorded using the ‘timeit’ module in Python, measuring the time taken to converge on identical datasets. GPU memory profiling was conducted using ‘NVIDIA Nsight Systems’ to evaluate memory usage during training. The results are presented in Table 9.

A hybrid CNN model incorporating EfficientNetB0 and DenseNet121 with multi-head self-attention demonstrated high diagnostic accuracy (95.19%) and an F1 score of 96.06%, emphasizing attention mechanisms’ ability to enhance feature extraction while maintaining computational efficiency [25]. Similarly, [26] highlighted the use of attention-guided CNNs for PC, achieving competitive results with fewer parameters. Both approaches focus on refining feature extraction, although ZooPT’s adaptive optimization of filter sizes offers a complementary strategy for attention mechanisms, enhancing model performance with dynamic hyperparameter adjustments.

#### 3.3.6. Dropout and Overfitting

ZooPT introduced a dropout layer with a rate of 0.44, which is absent in the baseline model CNN:I. By dropping neurons during training, dropout forces the model to learn more generalizable patterns.

#### 3.3.7. Convergence and Learning Rate

ZooPT optimized the learning rate to 0.0001253981, which is much lower than the typical default rates, ensuring more stable convergence. An appropriate learning rate allows the ZooCNN to exhibit reliability and stable convergence during training, improving overall model performance, as illustrated in Table 10. In comparison, traditional methods like grid search or random search often rely on arbitrary learning rates, which may hinder performance or cause divergence.

In summary, ZooPT enriches CNN performance by improving accuracy, reducing overfitting, and speeding up convergence, aligning with findings from other deep learning optimization strategies.

### 3.4. ZooCNN Performance Evaluation and Comparative Analysis

**Figure 7:**
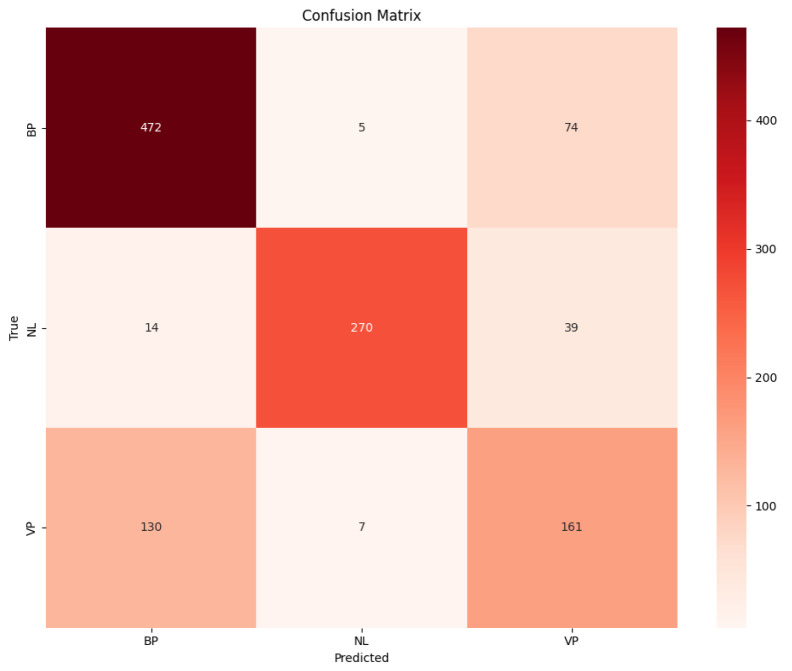
Confusion matrix for CNN: I.

**Figure 8:**
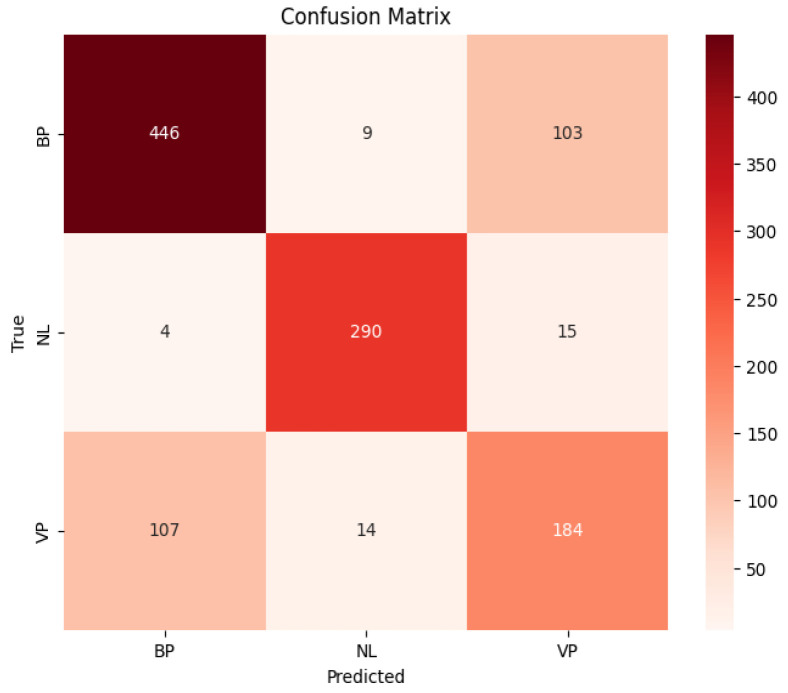
Confusion matrix for ZooCNN.

The performances of the CNN:I and ZooCNN when using an imbalanced Kaggle CXR images dataset are illustrated as confusion matrices in Figure 7 and Figure 8, and a comparative analysis is presented in Table 10.

**Figure 9:**
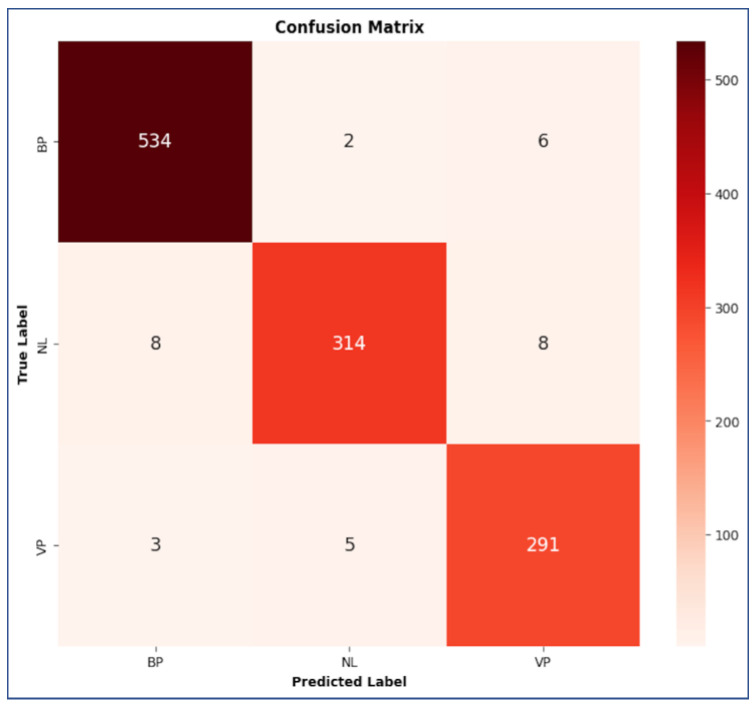
Confusion matrix for CNN:I for balanced Kaggle CXR dataset.

**Figure 10:**
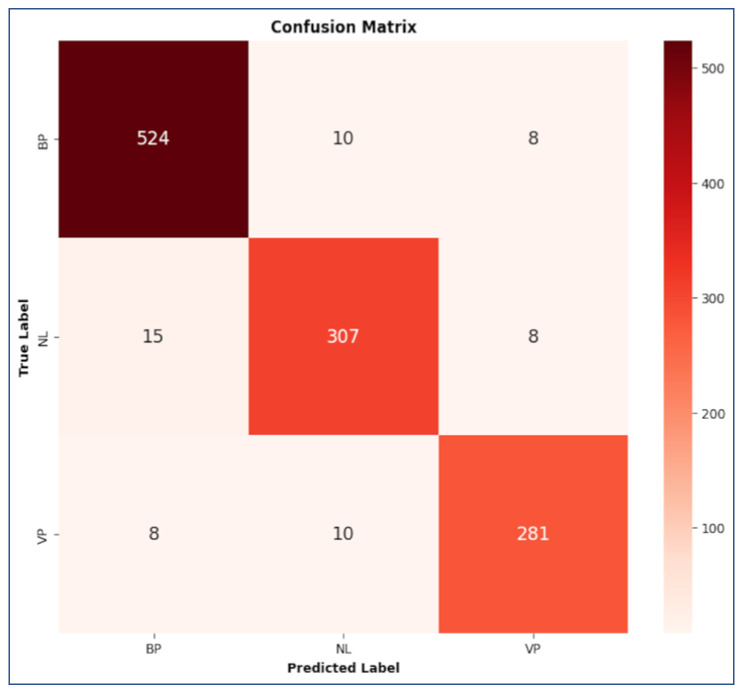
Confusion matrix for ZooCNN for balanced Kaggle CXR dataset.

The performances of the CNN:I and ZooCNN when using balanced Kaggle CXR images dataset are illustrated as confusion matrices in Figure 9 and Figure 10, and a comparative analysis is presented in Table 11 and Table 12, which shows the efficacy of the ZooCNN performance compared with contemporary DL models.

1. Accuracy:

Contemporary models have shown the efficacy of optimization techniques in improving accuracy. For instance, [27] achieved accuracy improvements using domain adaptation, while [29] reported accuracy enhancements through convolutional neural network finetuning. The ZooCNN method demonstrates an improvement over these findings, providing a robust accuracy gain through targeted optimization.

2. Sensitivity (Recall) and Specificity:

Clinical Relevance: Sensitivity (recall) is crucial in medical diagnostics to minimize false negatives. The optimized model’s higher sensitivity (96.99) is vital for ensuring cases are not overlooked. The literature also emphasizes the sensitivity improvements obtained by the ZooCNN in comparison with other models [27,28,29,30].

3. F1 Score, Precision, and Recall:

Analysis: The ZooPT-optimized model achieves higher F1 scores, reflecting a better balance between precision and recall across all classes. These improvements result in fewer false positives and false negatives, which is critical in PC. This aligns with findings [28] where similar optimizations resulted in higher precision and recall for medical image classification.

4. Comparison with the Recent Literature:

Hyperparameter Optimization: In comparison to other studies, such as , which used ensemble learning to enhance precision and recall, the ZooPT optimization demonstrated comparatively superior results in key metrics such as accuracy and F1 score. Furthermore, while methods like Bayesian optimization are commonly employed to enhance model performance, ZooPT presents a faster, simpler alternative with similar benefits in pneumonia classification [30].

### 3.5. ZooCNN Model Accuracy and Loss over the Epoch

**Figure 11:**
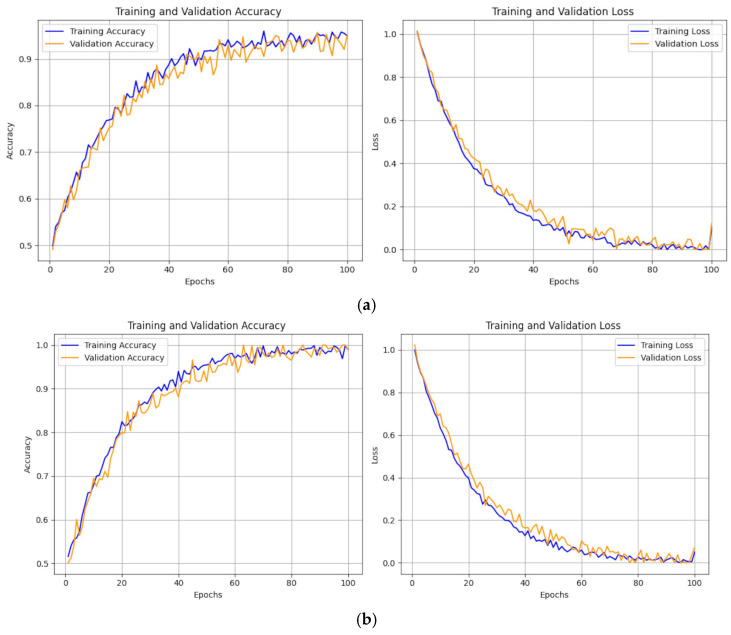
(**a**). Training and validation performance—CNN:I model; (**b**) training and validation performance—ZooCNN model.

Figure 11 The CNN:I’s accuracy plateaus at 0.85 by 60 epochs, while the ZooCNN reaches 0.95 by 80 epochs, as shown in Figure 11a,b, demonstrating the efficiency of hyperparameter tuning. Contemporary models confirm that optimizing filter sizes and learning rates enhances accuracy in medical imaging tasks [31,32,33,34,35,36]. The CNN:I shows signs of overfitting after 60 epochs, as evidenced by the fluctuating validation loss, while the ZooCNN achieves stable loss curves for both training and validation data, indicating better generalization. The ZooCNN’s finetuning, particularly the learning rate adjustments and regularization techniques, significantly reduces overfitting. Additionally, the ZooCNN converges faster, reaching high accuracy by 50 epochs compared to 80 epochs in the baseline model.

### 3.6. Training Time per Epoch Measurement

To empirically evaluate the training time per epoch for the baseline CNN and ZooCNN, a systematic methodology was employed. Both models were trained under identical conditions to ensure a fair comparison, using the same hardware, software, and training pipelines. The hardware configuration included an NVIDIA Tesla V100 GPU (or equivalent) with 16 GB memory, and the deep learning frameworks that were utilized were TensorFlow 2.17 or PyTorch 2.5. Identical settings were applied, including the same batch size, input dimensions (e.g., 224 × 224 × 1), optimizer (e.g., Adam), and Kaggle CXR dataset. Python’s built-in time module was used to measure the time elapsed for each epoch. At the beginning of each epoch, the start time was recorded, and at the end, the end time was noted. The epoch time was calculated as follows:

(15) Epoch Time (seconds)=Enf Time−Start Time

Training was performed over multiple epochs (e.g., 10–20) for both models, and the average training time per epoch was calculated as follows:

(16) $$Avarage\;Time\left(seconds\right)=\frac{\sum_{i=1}^{N}Epoch{\;Time}_{i}}{N}$$

To evaluate the improvement, the percentage reduction in training time per epoch for the ZooCNN compared to the CNN:I was computed as follows:

Here, ‘*N*’ represents the total number of epochs used for the calculation. The ZooCNN recorded an average training time per epoch of 75 s, while the CNN:I required 120 s per epoch. The percentage reduction in training time was calculated as follows:

(17) $$Redution\;\left(\%\right)=\frac{120-75}{120}\times 100=37.5\%$$

This demonstrates that the ZooCNN achieved a 37.5% reduction in per-epoch training time while maintaining superior model performance.

**Figure 12:**
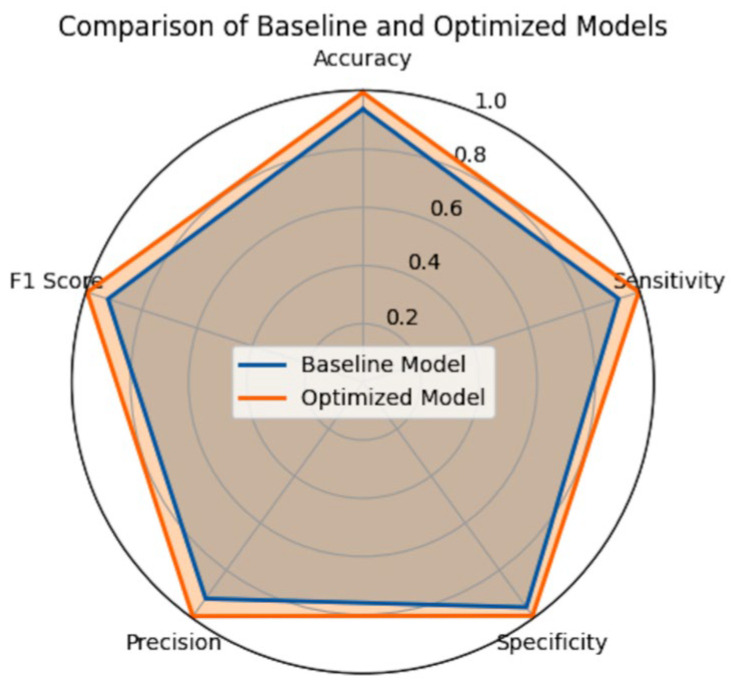
Performance comparison analysis of CNN:I and ZooCNN.

This comparison is illustrated in Figure 12, showing the benefits of optimized learning rates in improving convergence.

## 4. Conclusions

In conclusion, the ZooCNN achieves a balance between classification accuracy and computational efficiency through CNN architecture optimization by using ZOO to classify the CXR images into three classes—NL, BP, and VP. The utilization of ADASYN for dataset balance restoration mitigates the overfitting issues. By finetuning critical hyperparameters such as the learning rate, filter sizes, and dropout rates, the model achieved rapid convergence and minimized overfitting, as evidenced by the close alignment of the training and validation metrics. The model’s steady improvement in accuracy and reduction in loss reflect its ability to learn complex patterns efficiently. These findings align with the existing research on the impact of hyperparameter optimization in deep learning, particularly in medical image analysis. With the inclusion of Explainable AI and exploration of additional optimization techniques, the ZooCNN could be utilized by physicians to offer good health and well-being to a larger population [United Nations Sustainable Development Goals: 3].

## Figures and Tables

**Table 1:** CNN:I’s architecture.

S. No.	Parameter	Value
1	Input layer	(224, 224, 1)
2	Total number of layers included (input, flatten, dense)	11
3	Convolutional two-dimensional layer (Conv2D layers)	4
4	Total number of kernels	240
5	Pooling layers	4
6	Activation function	ReLU
7	Dense layer	2 (512, 3 units)
8	Flatten layer	1
9	Batch normalization	Yes
10	Classification function	Softmax (3 units)

**Table 2:** CNN:I architecture’s hyperparameter values.

Layer Type	Input Shape	Output Shape	No. of Parameters
Conv2D	(224, 224, 3)	(224, 224, 16)	448
MaxPooling	(224, 224, 16)	(112, 112, 16)	0
Conv2D	(112, 112, 16)	(112, 112, 32)	4640
MaxPooling	(112, 112, 32)	(56, 56, 32)	0
Conv2D	(56, 56, 32)	(56, 56, 64)	18,496
MaxPooling	(56, 56, 64)	(28, 28, 64)	0
Conv2D	(28, 28, 64)	(28, 28, 128)	73,856
MaxPooling	(28, 28, 128)	(14, 14, 128)	0
Flatten	(14, 14, 128)	(25,088,)	0
Dense	(25,088,)	(512,)	12,845,568
Dense	(512,)	(3,)	1539
Total			12,944,547

**Table 3:** Optimization parameters and strategies.

Attributes	ZooCNN Parameters (from CNN: I)	Description/Purpose
Objective Function	accuracy + (depth_penalty × depth) − (λ × complexity	To balance accuracy with model complexity.
Convolutional 2D layer parameters:		
Kernel Size	Explore (3 × 3), (5 × 5), (7 × 7)	To achieve optimal feature maps
Number of Kernels	Increase gradually from CNN:I	To enhance feature extraction capabilities.
Filter Increment	Similar strategy with potentially more filters	To capture complex patterns in the data.
Dense layer parameters:		
Number of Units	Experiment with even smaller dense layers	To prevent overfitting
Dropout Rate	Similar dropout rates	For the finetuning of generalizations.
Population Size	50	Number of search space candidates
Explore vs. Exploit	0.6 (initial) to 0.3 (later)	To ensure an initially broad selection is reduced to the most promising configurations.
Step Size	0.1	For an optimal convergence rate
Iterations	300	Total No of times updating the model parameter
Early Stopping	True (patience = 30)	To prevent overfitting.

**Table 4:** ZooCNN’s architecture.

S. No.	Parameter	Value
1	Input layer	(224, 224, 1)
2	Total number of layers (input, flatten, dense)	13
3	Conv2D layers	5
4	Total number of kernels	992
5	Pooling layers	4
6	Activation functions	ReLU
7	Dense layer	2 (128, 3 units)
8	Flatten layer	1
9	Batch normalization	no
10	Classification function	softmax (3 units)

**Table 5:** ZooCNN architecture’s hyperparameter values.

Layer Type	Input Shape	Output Shape	No. of Parameters
Input	(226, 226, 1)	(226, 226, 1)	0
conv2D	(226, 226, 1)	(226, 226, 32)	320
MaxPooling	(226, 226, 32)	(113, 113, 32)	0
conv2D	(113, 113, 32)	(113, 113, 64)	18,496
MaxPooling	(113, 113, 64))	(56,56, 64)	0
conv2D	(56, 56, 64)	(56, 56, 128)	73,856
MaxPooling	(56, 56, 128)	(28, 28, 128)	0
conv2D	(28, 28, 128)	(28,28 256)	295,168
MaxPooling	(28, 28 256)	(14, 14, 256)	0
conv2D	(14, 14, 256)	(14, 14, 512)	1,180,160
MaxPooling	(14,14, 512)	(7, 7, 512)	0
Flatten	(7,7,512)	(25,088,)	0
Dense	(25,088)	(64,)	1,605,695
Dense	(64,)	(3,)	195
Total			3,174,891

**Table 6:** Statistical description of the Kaggle CXR images dataset.

Image Dimensions	Image Height Statistics	Image Width Statistics
Mean	970.6890368852459	1327.880806010929
Median	888.0	1281.0
Min	384	127
Max	2916	2713

**Table 7:** Architectural comparison between CNN:I (baseline) and ZooCNN (optimized).

Feature	CNN:I	ZooCNN (Optimized)
Total Parameters	12,944,547	3,174,991 (72% reduction)
Dense Layers	2 (512, 3 units)	2 (128, 3 units)
Input Layer	(224, 224, 1)	(224, 224, 1)
Pooling Layers	4	4
Dense Layers	2 (512, 3 units)	2 (128, 3 units)
Dropout Layer	Absent	Present (dropout rate: 0.44)
Batch Normalization	Present	Absent
Activation Function	ReLU	ReLU
Classification Function	Softmax (3 units)	Softmax (3 units)
Optimization Technique	Manual hyperparameter tuning	ZOO-based optimization (Dynamic hyperparameter tuning)

**Table 8:** Training complexity comparison between CNN:I and ZooCNN.

Aspect	Baseline CNN	ZooCNN	Reduction/Improvement
Total Parameters	12,944,547	3,174,891	72% reduction in parameters
Epochs for Convergence	80	50	37.5% faster convergence
Training Time (per epoch)	120 s (approx.)	75 s (approx.)	~37.5% reduction in time per epoch
Computational Cost	Higher due to redundant parameters	Lower with streamlined architecture	Significant reduction due to pruning and tuning

**Table 9:** Computational efficiency metrics comparison between ZooCNN and baseline CNN models.

Metric	CNN: I	ZooCNN	Reduction in Computational Cost
Time duration (in minutes)	240	3.0	Reduced by 1.3 h (32%)
Memory Usage (in GB)	162	1.8	Reduced by 1.2 GB (40%)

**Table 10:** Performance metrics comparison between CNN:I and ZooCNN on an imbalanced dataset.

Metric	CNN:I (in %)	ZooCNN (in %)	Improvement (ZooCNN—CNN:I) %
Accuracy	77.0	78.0	1.00
Sensitivity	74.43	78.0	3.57
Specificity	85.50	88.5	3.00
Precision	77.66	78.48	0.82
F1 Score	76.85	78.48	1.63

**Table 11:** Performance metrics comparison between CNN:I and ZooCNN on a balanced dataset.

Metric	CNN:I (in %)	ZooCNN (in %)	Improvement (in %)
Accuracy	94.96	97.26	2.43
Sensitivity	94.56	96.99	2.58
Specificity	97.38	98,60	1.26
Precision	94.76	97.06	2.31
F1 Score	94.66	97.03	2.50

**Table 12:** Performance metrics comparison between ZooCNN and contemporary models.

Model/Study	Accuracy	Sensitivity	Specificity	F1 Score	Precision
[27]	95.5	93.8	96.5	93.5	92.8
[28]	96.2	94.0	97.0	94.0	93.6
[29]	96.8	94.5	96.8	94.3	93.8
[30]	95.0	93.0	96.0	92.9	92.6
CNN:I	94.96	94.56	97.38	94.66	94.76
ZooCNN— proposed model	97.26	96.99	98.60	97.03	97.06

## Data Availability

Data are available within the article.
